# Recommendations on how to ensure the safety and effectiveness of biosimilars in Latin America: a point of view

**DOI:** 10.1007/s10067-015-2887-0

**Published:** 2015-02-12

**Authors:** Carlos Pineda, Carlo V. Caballero-Uribe, Marcia Gonclaves de Oliveira, Pedro Saul Lipszyc, Jose Julian Lopez, Marcelo Mario Mataos Moreira, Valderilio Feijo Azevedo

**Affiliations:** 1 Research Direction, Instituto Nacional de Rehabilitación, Mexico City, México; 2 Universidad del Norte, Barranquilla, Colombia; 3 ANVISA, Brasília, Brazil; 4 Department of Pharmacology, Medical School, University of Buenos Aires, Buenos Aires, Argentina; 5 Centro de Información Medicamentos de la Universidad Nacional (CIMUN), Bogotá D.C., Colombia; 6 Federal University of Parana, Curitiba, Brazil

**Keywords:** Biosimilars, Naming, Pharmacovigilance, Surveillance, Traceability

## Abstract

The use of biotechnology-derived medicines has significantly increased in recent decades. Although biosimilars undergo rigorous characterization as well as clinical studies to document their safety and effectiveness, they are highly complex molecules and small changes in the purification and production process of a biosimilar can have major implications in its safety and effectiveness profile. In Latin America, regulatory authorities have begun to establish well-described and standardized pathways that permit a biosimilar to gain commercial licensure. In order to be certain that a biosimilar reaches its potential in ordinary clinical use, an intensive post-licensing monitoring system must be established since it is the only means to ascertain the true similarity between the original biologic and its biosimilar. Pharmacovigilance allows national authorities to determine a drug’s performance in the marketplace. An effective tracking and pharmacovigilance system for biological medicines has many steps and processes. To aid policy makers in Latin American in addressing the many issues surrounding the establishment of an effective pharmacovigilance system, the Americas Health Foundation convened a group of experts to discuss the topic and develop recommendations for implementation. The group discussed current challenges and gaps in pharmacovigilance in Latin America, paying close attention to the major issues associated with traceability and pharmacovigilance of biosimilars following their approval. The recommendations developed should enable countries to accurately document the safety and performance of a biosimilar as experienced by patients under real-life conditions and have a significant impact on the successful implementation of pharmacovigilance of biosimilars throughout the region.

## Introduction

The use of biotechnology-derived medicines has increased dramatically in recent decades. As these products have come off patent protection, presumed biochemical and therapeutically equivalent products have been developed and have been termed biosimilars [1]. That is, a biosimilar is a biologic product that can be expected to produce the same therapeutic effect in any given patient had the original, or innovator, biologic medicine been given. Thus, biosimilars are considered to be similar in efficacy and safety to their reference products and as such are gaining widespread acceptance as suitably interchangeable medicines [2].

Although biosimilars undergo rigorous characterization as well as clinical studies to document their safety and effectiveness, the fact remains that they are highly complex biological molecules. Small changes in the production and purification process of a biological can have major implications in its safety and effectiveness profile [3]. Therefore, not only is thorough pre-clinical and clinical testing critical but demonstrating the true similarity of a biosimilar to its original product also requires careful documentation in post-marketing studies. The ability to trace and track a medicine introduced into the marketplace is essential. When given to large numbers of patients, it is not uncommon to document safety issues that were not seen in the controlled trials necessary for licensure. This is particularly important for biosimilars because of their multi-faceted manufacturing processes and because, as stated above, it is not unreasonable to expect that there may be subtle differences in composition between a biosimilar and its reference product. Such differences may be revealed in an altered safety and effectiveness profile that is only detected when the medicine gains widespread use.

In Latin America, regulatory authorities have begun to establish well-described and standardized pathways that allow a biosimilar to gain commercial licensure [1, 4]. In order to be certain that a biosimilar achieves its potential in ordinary clinical use, an intensive post-licensing monitoring system must be established since it is the sole means to ascertain the true similarity between the original biologic and its biosimilar. Unfortunately, few Latin American countries have such systems actively working to capture the relevant information.

Pharmacovigilance (also known as surveillance) allows national authorities to determine a drug’s performance in the marketplace. The first component of an effective pharmacovigilance system is the ability to track the medicine from manufacture to distribution to prescription. Then, there must be a system of pharmacovigilance that encourages and is able to document all adverse events that may be associated with the use of the medicine. Finally, the data derived from such reports must be carefully analyzed and reported to health professionals as well as to the manufacturer [5–7].

An effective tracking and pharmacovigilance system for biological medicines has many steps and processes. To aid policy makers in Latin American countries in addressing the major issues surrounding the establishment of an effective pharmacovigilance system, the Americas Health Foundation convened a group of experts to discuss the topic and develop recommendations for implementation. The group discussed the current challenges and gaps in pharmacovigilance in Latin America, paying close attention to the major issues associated with the traceability and pharmacovigilance of biosimilars following their approval. The recommendations presented below should enable countries to document accurately the safety and performance of a biosimilar as experienced by patients under real-life conditions.

## Naming

The name of the product is the foundation of product identification and is therefore absolutely essential for accurate record keeping and the subsequent attribution of adverse events. Currently, under the WHO’s International Nonproprietary Name (INN) policy, the INN for a new biosimilar may be the same as that of the original biologic medicine. In such a case, if only the INN, without a distinguishable identifier, is used when prescribing a biologic medicine, an adverse event may be difficult to attribute to a specific product. It would then be unclear what medicine caused the adverse event, leading to a diminished ability to document long-term product safety [8, 9].

There is a sharp divide on the issue of the name given to a biosimilar with some stakeholders calling for biosimilars to be assigned the same INN as their reference biologic. These individuals believe that a biosimilar is equivalent to a generic drug and thus want biosimilars treated as generics, which means they would have the same INN as the reference product. Others suggest the use of distinct propriety names or nomenclature. Their claim is that a biosimilar is not, and cannot be, identical to the reference product and thus requires a unique identifier. Moreover, looking toward the day when multiple biosimilars are related to one reference product, the use of identical INNs could lead to inadvertent switching at the pharmacy and the inability to establish the root cause of any adverse effects associated with a particular medicine. Faced with a unique INN, however, the inadvertent switching of products between patients at pharmacies can be avoided, thereby improving traceability and improving pharmacovigilance.

The use of distinct brand names may help to distinguish between products. All biosimilar products in the European Union have a distinct brand name, and many stakeholders have suggested that the use of distinct brand names for biosimilars will eliminate the need for distinct non-proprietary names [10]. However, not all jurisdictions have the authority to require that products bear a brand name. For example, in the USA, FDA does not have explicit statutory authority to require that all biosimilars have a distinct brand name. In some Latin America countries (e.g., Colombia), clinicians are obligated to prescribe drugs in the public health system using only the INN. In Brazil, for example, prescriptions may not have brand names in the SUS (Sistema Unico de Saúde). Thus, the use of distinct non-proprietary names becomes critical to distinguish biosimilars from their reference products. In summary, either a distinct brand name or a distinct non-proprietary name is crucial in order to implement effective pharmacovigilance.

## Traceability

Biosimilars are not generic products. Unlike with generics, there may be subtle differences between biosimilars from different manufacturers or a biosimilar compared to its reference product. To differentiate between medicines, an adverse event must be traceable to a specific biological product. Thus, clearly identifying the specific biological product being given to a patient is critical to pharmacovigilance monitoring.

Traceability is a set of procedures that follows the manufacture, distribution, and prescription of medicines throughout the supply chain by means of a well-documented identification system. While traceability is often not considered part of a pharmacovigilance program, in fact, effective traceability strategies are essential to improved pharmacovigilance. Thus, traceability is critical to protecting patient safety because health authorities need accurate data linking adverse events to specific medicines [11]. As awareness of the benefits of having an accurate and complete tracing system has become apparent, efforts have been directed toward making traceability a regulatory requirement and developing approaches to enable and facilitate the implementation of traceability. Ultimately, the traceability of biosimilars is essential to satisfying basic clinical and public health needs. On the subject of traceability, four main issues are critical: (1) naming, (2) record keeping, (3) recall mechanisms, and (4) the drug origin. Of these, naming has been the most controversial and unclear.

## Pharmacovigilance

Pharmacovigilance is the science of collecting, monitoring, assessing, and evaluating information from healthcare providers and patients on adverse effects or any other drug-related problem to protect patient safety. The overall goal of post-marketing is to accurately and promptly trace a patient’s adverse event to a particular product and manufacturer [7]. Proper labeling, product tracking and a standardized operational system of pharmacovigilance, and attributing adverse events are all components of a well-functioning pharmacovigilance program.

The main issues involved in monitoring the safety and effectiveness of a commercial pharmaceutical are as follows:

- Identifying the patient receiving the drug. Monitoring the patient over time for adverse reactions and the effectiveness of the product.
- Substitution or interchangeability
- Extrapolation of indications
- Data collection
- Data analysis
- Data reporting

The prescription itself serves as a record of who receives a drug, the dose and frequency of administration and potentially the indication for its use. Therefore, the prescription itself can serve as the initial step in a pharmacovigilance program. When an adverse event occurs, it is then easier for a physician to relate the event to a specific drug.

## Substitution

Substitution occurs when a drug is dispensed that is not the drug prescribed. In other words, in the case of biologics, substitution is construed as the legal authority for a hospital or a pharmacy filling a prescription to switch from dispensing the innovator product to a biosimilar or the reverse. Substitution of generic drugs for reference drugs is uncontroversial because the two will be identical if they have demonstrated bioequivalence. However, the issue becomes more complex when considering the substitution of biosimilars. While biosimilars are similar to the originator drugs, as mentioned above, they are not identical and therefore there is currently no scientific basis to substitute different products. At this time, regulatory decisions concerning substitution are currently left to individual countries [12]. For example, the EMA does not have the authority to designate a biosimilar as automatically substitutable. The recent trend in Latin America is toward the adoption of accepted international standards [13].

A lack of clear guidelines on this issue and the consequence of substitution of one biological medicine for another can severely impact patient safety and make post-marketing pharmacovigilance more difficult. The issue of substitution is also closely tied to naming because if doctors prescribe biologics by a unique identifier, rather than the currently used INN, substitution of a biosimilar product when dispensed by a pharmacist would likely occur much less often. Also, in Latin America, another concern is related to adverse effects that may occur when patients are switched back and forth between the innovator and the biosimilar or even the intended copy [14].

Biosimilars are considered interchangeable if a therapeutically equivalent biologic can be prescribed by a physician in place of its reference product [15, 16]. According to the FDA’s Biologics Price Competition and Innovation Act (BPCIA), “to meet a higher standard of “interchangeability,” an applicant must provide sufficient information to demonstrate biosimilarity, and also to demonstrate that the biological product can be expected to produce the same clinical result as the reference product in any given patient and, if the biological product is administered more than once to an individual, the risk in terms of safety or diminished efficacy of alternating or switching between the use of the biological product and the reference product is not greater than the risk of using the reference product without such alternation or switch..”

## Extrapolation of Indication

In some circumstances when a biologic has been shown to be similar to its reference product, and approved for the same indication, some experts allow an extrapolation of indication to those of the reference product. This seems acceptable if the other diseases for which the reference product is indicated are truly similar. Also, if clinical similarity can be shown in a key indication, extrapolation of efficacy and safety data to other indication(s) of the reference product may be possible, if the relevant mode of action and/or the receptor(s) involved in the extrapolated indications are the same.

## Data Collection, Analysis, and Reporting

One of the primary weaknesses of current pharmacovigilance programs is that they are based on sparse and incomplete reporting. This does not allow for proper ascertainment of the total number of people exposed to the drug and the rate and nature of adverse events, making it difficult to estimate the magnitude of risk and to analyze the root causes of adverse drug reactions. Pharmacovigilance should go beyond adverse drug reaction reporting because drugs should also be monitored for their effectiveness (i.e., some drugs have been withdrawn due to poor risk-benefit ratios). Moreover, in addition to biosimilars, some reference products have also been associated with adverse drug reactions, which necessitate their inclusion in any intensive pharmacovigilance program.

The development of a pharmacovigilance system should be based on risk balancing strategies rather than simply reacting to adverse events. A Risk Management Plan (RMP) [17], developed by the manufacturer, increases the likelihood that the benefits of a medicine exceed its risks by the greatest achievable margin.

A RMP is a set of activities and interventions designed to identify, characterize, and manage the risks relating to the use of a medicine. It consists of the following:

- an overview of the safety profile of the medicine
- a pharmacovigilance plan, and
- a risk-minimization plan.

Thus, the RMP is a document describing the risk management of a drug from its development to its commercial use. In addition, the RMP aims to evaluate the drug at regular intervals (e.g., every 6 months) or in response to post-marketing pharmacovigilance reports. In submitting adverse event reports to the manufacturer and/or national authorities, health professionals should ensure that they clearly identify the product suspected to have caused the adverse event. Since the RMP covers the entire life cycle of the product, it should be periodically updated to reflect new knowledge and understanding of the safety profile of the product. Sharing the information among medical professionals is meant to ensure further enhancements of post-marketing safety measures. RMPs also include information on the following:

- a medicine’s safety profile,
- how its risks will be prevented or minimized in patients,
- plans for studies and other activities to gain more knowledge about the safety and efficacy of the medicine,
- risk factors that are related to side effects, and
- measuring the effectiveness of risk-minimization measures.

## Recommendations

- Naming:
  - Regulatory authorities should consider adopting non-proprietary names or unique identifiers for biosimilars.
- Traceability:
  - Pharmacovigilance legislation should stress the importance of traceability, and all countries in Latin America should implement activities to improve traceability [11, 18].
  - Given the importance of accurate identification of prescribed biologics and biosimilars, in order to support pharmacovigilance monitoring, all medicinal products must be clearly defined by the use of a specific identifier.
  - Each biosimilar should have a unique identifier that is standard across the region. One approach is to use the manufacturer’s name in order to differentiate the biosimilar from the reference product.
  - The unique identifier should be included in adverse reaction reporting.
- Substitution and Interchangeability:
  - If a product is interchangeable with another, the implication is that both have been shown to have similar safety and effectiveness. If that’s the case, substituting one for another is acceptable. If, however, there is some doubt about their similarity, then only a physician should authorize substitution of one product for another.
- Data Collection, Analysis, and Reporting:
  - The pharmacy should report all dispensing information to national authorities. The national authority should collect at least the following information in each prescription *for biologics* being filled by pharmacies:
    - Name of the drug
    - Dose and frequency
    - Treatment duration
    - Patient identifier
  - All potential drug side effects or adverse effects should be reported to national authorities by a health professional and all reported data should go to the national pharmacovigilance/pharmacovigilance agency
  - National pharmacovigilance authorities should evaluate data received on a regular basis and analyzed data (specifically pertaining to adverse events) should be publicly reported on a regular basis
  - In order to facilitate the reporting of data regarding biological medicines to national pharmacovigilance agencies:
    - Online reporting should be established
    - Medical schools should emphasize the importance of pharmacovigilance, which should be supported by national societies.
    - Governments should actively and regularly promote pharmacovigilance
- Currently, most countries in Latin America have multiple agencies or organizations receiving adverse event reports. These multiple systems should be integrated into one national pharmacovigilance system so that there is a single database in which all adverse events are captured [19].
- All Latin American countries should establish a certification program in order to train pharmacovigilance experts. Such experts would be trained to manage all aspects of a country’s data collection and/or analysis related to the use of pharmaceuticals. The successful implementation of new and better pharmacovigilance systems requires that health professionals and regulatory agencies be trained in the need, value, and operation of the country’s “pharmacovigilance system.” This requires continuing education about pharmacovigilance processes and procedures, as well as changing behavior by increasing awareness of the importance of pharmacovigilance.
- The development of a Risk Management Plan should be mandatory for all biological products.
